# Titanium Dioxide/N-Doped Graphene Composites as Non-Noble Bifunctional Oxygen Electrocatalysts

**DOI:** 10.1021/acs.iecr.1c02896

**Published:** 2021-11-19

**Authors:** José Manuel Luque-Centeno, María Victoria Martínez-Huerta, David Sebastián, Sara Pérez-Rodríguez, María Jesús Lázaro

**Affiliations:** † Instituto de Catálisis y Petroleoquímica (CSIC), Marie Curie 2, 28049, Madrid, Spain; ‡ Instituto de Carboquímica (CSIC), Miguel Luesma Castán 4, 50018, Zaragoza, Spain

## Abstract

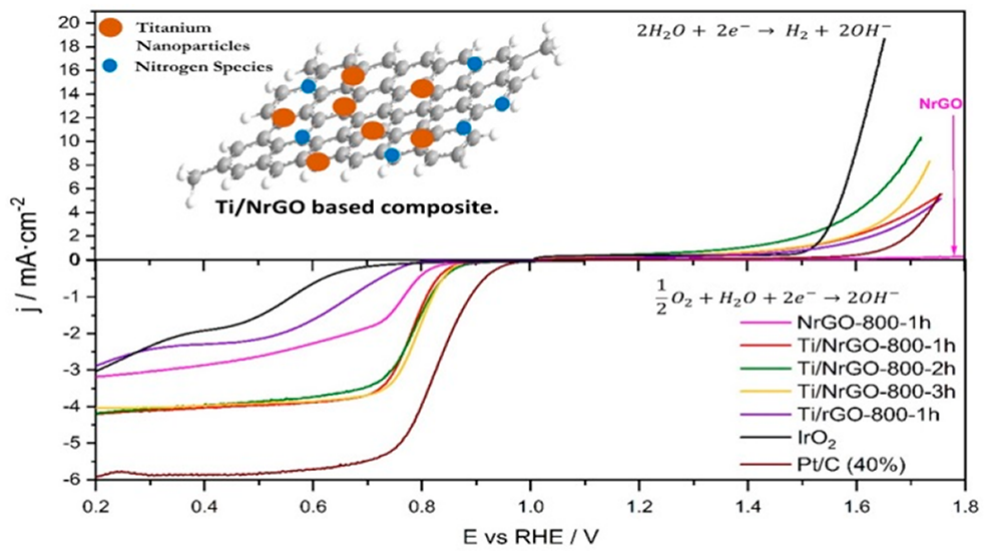

Bifunctional oxygen electrocatalysts are essential in the development of low-temperature unitized regenerative fuel cells (URFCs), as a promising alternative for storing energy via hydrogen. TiO_2_, as a semiconductor material, is commonly not established as an active electrocatalyst for oxygen reduction and oxygen evolution due to its poor electrical conductivity and low reactivity. Here, we demonstrated that composites composed of TiO_2_ and N-doped graphene can be active in oxygen reduction and evolution reactions in an alkaline environment. Combination factors such anatase/rutile interaction, N-doping graphene, and the presence of Ti^3+^/Ti–N species raise the active sites and improve the electrochemical activity. Our results may afford an opportunity to develop a non-noble and promising electrocatalyst in energy storage technology.

## Introduction

Climate neutrality is one of the key objectives of the European Union, proposing a transformation of the energy system before the year 2050. Hydrogen produced from renewable energy sources is positioned as one of the main energy carriers in the long term because its production and consumption is climate neutral and does not generate polluting emissions.^1^ Low-temperature unitized regenerative fuel cells (URFC) are a promising alternative for storing energy via hydrogen. A URFC consists of a single device capable of working as a fuel cell (FC) and in water electrolysis (WE), in such a way that only one of the modes is operational in time. Hydrogen is generated by the electrolysis of water and is stored and used directly in the URFC to obtain electricity when necessary.^2,3^ By comparison with other conventional energy storage technologies, URFC displays benefits such as simple system design, high energy storage capacity (theoretically up to 3660 Wh kg^–1^), and eco-friendliness.^4,5^

The URFC technology market is hampered by the excessive price of electrocatalysts due to the sluggish kinetics at the oxygen electrode toward the oxygen reduction reaction (ORR) and the oxygen evolution reaction (OER), which involves a four-electron transfer process.^5^ Electrocatalysts are essential to providing high device efficiency by decreasing the overpotential losses for each reaction.^6^ Therefore, bifunctional oxygen electrocatalysts are crucial in the development of URFCs. However, the development of a bifunctional catalyst is not obvious. For the ORR, the best catalytic materials known are Pt-based, which are not active for OER. In the other way, Ru and Ir based electrocatalysts are good for the OER but are not active for ORR.^7,8^ Alkaline anion exchange membranes open up the possibility of noble metal-free catalysts, since the oxygen reactions are more favorable from a kinetics viewpoint than with acidic membranes.^4^ In the past few years, several studies have demonstrated that transition metals combined with carbon materials can be employed as good bifunctional catalysts, showing good catalytic performance for both reactions and stability in alkaline media.^9−13^

Titanium oxide is an earth-abundant, low cost, highly stable, and environment-friendly material. However, this semiconductor material is electrically insulating at temperatures under 200 °C, causing less interest in TiO_2_ as an ORR/OER electrocatalyst despite its advantages. Remarkable efforts have been made to induce electronic conductivity for improving TiO_2_ electrochemical properties. The nonstoichiometric reduction of TiO_2_ improves donor density and electrical conductivity as well as the overall electrocatalytic performance, due to the incorporation of structural defects, i.e., “oxygen vacancies” (Vo) and Ti^3+^.^14−17^ Pei at al. demonstrated, by combining electrochemical tests with density functional (DFT) calculations, that nanostructured TiO_2_, self-doped by oxygen vacancies and selectively exposed with high energy {001} facets, revealed unexpectedly competitive ORR activity, outstanding stability, and superior methanol tolerance.^14^

To date, few research works have been reported on the use of TiO_2_ as an active site for ORR and OER in an alkaline medium.^14,18−22^ Most of them develop a similar strategy using carbon-based materials to increase the activity of titanium oxide. For ORR, Boppella et al. reported conductive TiO_2_ attached on reduced graphene oxide (rGO) hollow nanospheres.^18^ They found a significant enhancement of the TiO_2_ conductivity with an improved activity and stability toward ORR. The results were ascribed to a cooperative effect of the hybridization of TiO_2_ with reduced graphene oxide, Ti^3+^ self-doping, and the development of a carbon-coating layer over the TiO_2_ particles. On the other hand, the incorporation of N atoms into the carbon matrix can also benefit the electrochemical activity. Jin et al. developed a thermolytic method for the preparation of N-doped TiO_2_/nanoporous carbon hybrid materials, which allowed thermal control over the anatase/rutile ratio and the nitrogen incorporation.^20^ The best performance for the ORR was obtained with an optimal composition of 5 atom % N-doping with anatase phase content of 5 mol %. For the oxygen evolution reaction, Shan et al. prepared electrodes containing carbon, oxygen, and titanium (NanoCOT) with high efficiency to OER performance with a low overpotential.^21^ They found predominant valence and defect states of Ti (Ti^1+^, Ti^2+^, Ti^3+^, and Ti^4+^) on the TiO_2_ surface and substantial hybridization of the C 2p and O 2p states, which shows a significant role in enhancing electronic conductivity and activity. A comprehensive study on the effect of anatase and rutile phases on OER in 1 M KOH was carried out by Hu et al.^22^ They synthesized TiO_2_/rGO nanocomposites by tuning the rutile/anatase ratio. The composite with a similar loading of rutile and anatase phases adsorbed the most hydroxyl species and showed the best OER performance. Electrocatalysts based on TiO_2_ distributed in a N-doped matrix exhibited robust trifunctional electrocatalytic activity toward the hydrogen evolution reaction (HER), oxygen evolution reaction (OER), and oxygen reduction reaction (ORR).^19^ They suggested that the combination of anatase phase with the N-doped carbon was important for the ORR activity.

In this work, composites formed by titanium oxide and N-doped reduced graphene oxide (NrGO) were prepared and investigated as bifuntional electrocatalysts for the ORR and the OER under alkaline conditions. Our previous study established that titanium oxide can be active in both reactions.^23^ To elucidate the promotion effect, in this work, the influence of the nitrogen atom and the annealing time at 800 °C in the composite synthesis have been studied. Electrocatalysts were characterized in order to find out correlations between their catalytic behavior and their structures.

## Experimental Section

### Chemical and Materials

The commercial graphite powder with high purity (>99.8%) and a particle size below 20 mm, urea (>98%), KMnO_4_ (>99.8%), NaOH (99.99%), and Nafion (5 wt %) were provided by Sigma-Aldrich. H_2_SO_4_ (96%) was purchased from Merck. H_2_O_2_ (33% w/v), titanium(IV) *n*-butoxide (>99%), IrO_2_ (99%), and ethanol (96%) were acquired from Panreac. The commercial catalyst, Pt/C (40 wt %), was purchased from Johnson Matthey. All of the chemicals were used as received without further purification. Ultrapure water with a resistivity ≥18 MΩ cm was obtained through the Millipore system (Milli-Q) in all of the experiments.

### Catalysts Synthesis

The graphene oxide (GO) was prepared by a modified Hummers’ method.^24^ The synthesis of the composites was carried out typically as follows: GO was dispersed in ethanol by ultrasonication, and then titanium *n*-butoxide was added slowly to the solution. The mixture was stirred for 30 min, sonicated for additional 30 min, and, after that, an appropriate amount of urea was added to the dispersion, which was stirred until urea was completely solubilized. The metal to urea molar ratio was kept at 1:20. This dispersion was left overnight to end the gel formation. Finally, the resultant gel was transferred into a quartz tubular reactor and annealed at 800 °C for different durations (1 h, 2 h, 3 h). In order to remove impurities, the final composites were thoroughly washed with water and acetone. The resulting composites were labeled as Ti/NrGO-800-1h, Ti/NrGO-800-2h, and Ti/NrGO-800-3h. In order to individuate the influence of Ti and N in the catalysts, two more samples, one without Ti and one without N, were also synthesized with 1 h of annealing time and labeled as NrGO-800-1h and Ti/rGO-800-1h.

### Physicochemical Characterization

Titanium loading was determined by inductively coupled plasma and optical emission spectroscopy (ICP-OES) using a SPECTROBLUE AMETEK spectrometer. The elemental analysis (C, N) was carried out using a Thermo Flash 1112 analyzer. X-ray powder diffraction (XRD) measurements were performed on a Bruker D8 Advance Polycrystalline Powder X-ray Diffractometer with a Cu Kα source. The average crystallite sizes for TiO_2_ phases and graphene (*L*_c_) were obtained by fitting the diffraction patterns applying the Pawley or LeBail algorithm using the software TOPAS. The cell lattice parameters were refined to pseudo-Voigt functions for anatase and rutile-TiO_2_ and the Split Pearson VII model for graphite. The distribution and valence state of elements in the near surface layer were determined by X-ray photoelectron spectroscopy (XPS) with an OMICRON ESCA p spectrometer with a dual X-ray source (MgKα 1/4 1253.6 eV, AlKα 1/4 1486.6 eV). CasaXPS software was used for calculating atomic percentage compositions, using Gauss-Lorentz equations with the Shirley-type background. To convolute the high-resolution spectra, a 70%/30% Gaussian/Lorentzian line shape was used. Raman spectra were obtained with a Renishaw in Via Raman Microscope spectrometer equipped with a laser beam emitting at 532 nm and 5 mW output power. The morphology and the particle size were analyzed in a Tecnai F30 high resolution transmission electron microscope (TEM) operating at an accelerating voltage of 200 kV.

### Electrocatalytic Measurements

Electrochemical procedures were carried out in a three-electrode cell controlled by a potentiostat/galvanostat AutoLab workstation (PGSTAT302N), using a high surface glassy-carbon rod as a counter electrode and a reversible hydrogen electrode (RHE) in the supporting electrolyte as a reference electrode. A rotating disk electrode (RDE) with a glassy carbon disk of 5 mm diameter (area = 0.196 cm^2^) or a rotating ring disk electrode (RRDE) with the same disk characteristics and a Pt ring were used as a working electrode (WE). The WE was prepared by loading 30 μL of catalytic ink (prepared by sonicating 4 mg of catalyst with 15 μL of Nafion and 385 μL of a mixture of isopropanol (IPA) and ultrapure water (IPA:H_2_O, 3:2)) on the glassy carbon electrode. The supporting electrolyte was a 0.1 M NaOH aqueous solution. For all measurements, N_2_ (99.99% Air Liquide) was employed to deoxygenate the electrolyte. The ORR experiments were carried out in O_2_ (99.995% Air Liquide) saturated alkaline solution. The catalysts were submitted to an initial activation process based on 50 cyclic voltammograms (CVs) between 0.05 and 1.2 V vs RHE at a scan rate of 0.1 V s^–1^ in deoxygenate supporting electrolyte. The ORR activity was performed by a polarization curve between 1.0 and 0.05 V vs RHE (negative going scan) with a sweep rate of 0.005 V s^–1^. Before the ORR polarization curves, all samples were kept at 1.0 V for 60 s. The ORR kinetics were investigated using different rotating rates (rpm) to calculate the number of electrons by Koutecký-Levich plots. The analysis was performed maintaining the ring at 1.2 V vs RHE in order to detect hydrogen peroxide formation. The OER activity was performed using a polarization curve between 0.7 and 1.8 V vs RHE (positive going scan) at 0.005 V s^–1^ and 1600 rpm, keeping the ring at 0.4 V vs RHE (for quantification of the evolved O_2_ by ORR).^13^ In addition, potential values were *iR*-corrected considering the series resistance (40 Ω), which was determined by electrochemical impedance spectroscopy at the open circuit potential and a high frequency (EIS).

### Structure, Composition, and Morphology of the Composites

The bulk chemical composition of the synthesized electrocatalysts was determined by elemental analysis and ICP-OES (Table 1). Chemical analysis showed the effective titanium deposition and nitrogen incorporation into the structure of reduced graphene oxide. The ICP-OES results revealed an increase of the titanium loading with the annealing duration from 19 wt % for the sample subjected to 800 °C for 1 h (Ti/NrGO-800-1h) to 29 wt % for the counterpart treated for 3 h (Ti/NrGO-800-3h). Regarding the nitrogen content, the thermal treatment at 800 °C for 1 h was the most effective for the intercalation of nitrogen (11 wt %). Longer annealing treatments led to a substantial decrease in the amount of nitrogen with values of 7 and 2 wt % for Ti/NrGO-800-2h and Ti/NrGO-800-3h, respectively. This dependence of the nitrogen content on the annealing time is also evident by comparing the similar nitrogen contents of the samples subjected to heat treatment for 1h: Ti/NrGO-800-1h (11 wt %) and its metal-free analogous (NrGO-800-1h, 12 wt %). The latter also suggests that the presence of titanium during the formation of the composite does not present any influence on nitrogen doping.

**Table 1:** Chemical Composition of the Composites from Elemental Analysis (C, N) and ICP-OES (Ti)

material	C (wt %)	N (wt %)	Ti (wt %)
Ti/NrGO-800-1h	56	11	19
Ti/NrGO-800-2h	54	7	22
Ti/NrGO-800-3h	33	2	29
Ti/rGO-800-1h	51		25
NrGO-800-1h	86	12	

**Figure 1:**
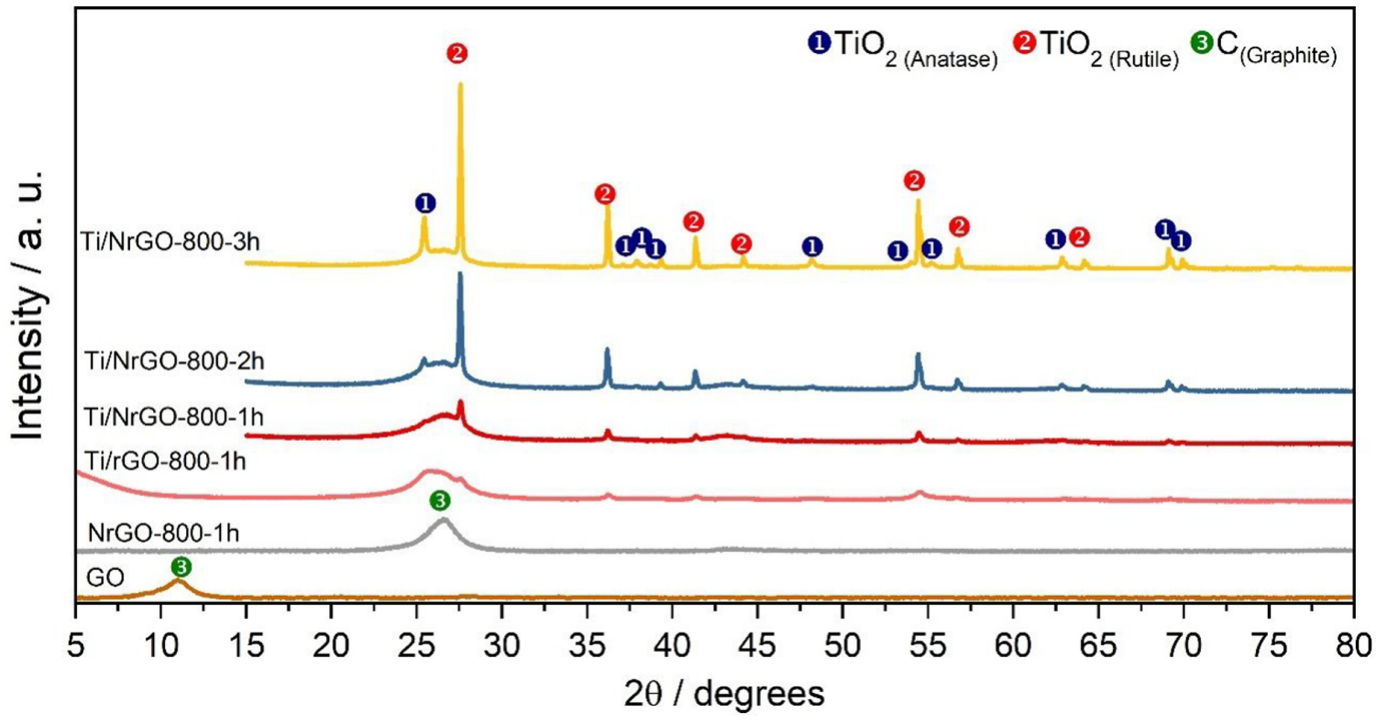
XRD patterns of composites, NrGO-800-1h and GO. The diffraction lines were normalized by the signal associated with the graphite (002) basal plane (2θ = 26.5°).

The XRD patterns of the composites obtained at different annealing times compared to those of NrGO-800-1h, Ti/rGO-800-1h, and GO are depicted in Figure 1. GO presents a diffraction peak at 2θ = 10.8° related to the larger interlayer spacing graphene oxide (*c*/2) by the intercalation of oxygenated species among the graphenic layers. As a result of the thermal reduction at 800 °C during the synthesis of the composites and the Ti-free material (NrGO-800-1h), the diffraction (002) peak shifted to higher Bragg angles (c.a. 26.5°) due to the removal of oxygenated species and the consequent shrink of interlayer distance (*c*/2).^25^Table S1 shows the values of the mean crystallite size for carbon (*L*_c_) and the average number of graphene layers (*N* = *L*_c_/(*c*/2) + 1). The composites obtained at different annealing times exhibited similar *L*_c_ values ranging from 2.1 to 3.3 nm and with a number of graphene layers of 7–11, which shows a negligible effect of heat treatment duration on the restacking of graphene layers.

Regarding the titanium phases, XRD patterns of all synthesized composites exhibit seven peaks at 2θ = 27.5°, 36°, 41.3°, 44.1°, 54.3°, 56.5°, and 64° attributed to the (110), (001), (111), (120), (121), (220), and (002) crystallographic planes of the TiO_2_-rutile phase (JCPDS No. 89-4202), respectively.^13,23,26−29^ The characteristic peaks of TiO_2_-anatase can also be observed for all TiO_2_-based electrocatalysts at 25.3°, 37.0°, 37.8°, 38.5°, 48.0°, 54.0°, 55.0°, 62.7°, 68.9°, 70.3°, and 75.1° (JCPDS No. 89-4921),^22,29−31^ with the exception of the diffraction pattern of Ti/NrGO-800-1h. Moreover, the peaks corresponding to both TiO_2_-anatase and rutile phases present a higher relative intensity as the duration of the annealing treatment increases, confirming a larger crystalline domain. The latter is more evident in Table S1, which summarizes the values of the crystallite sizes of TiO_2_ phases. The crystallite size of the rutile phase rises to 2 times after pyrolysis for 3 h compared to its analogous treatment for 1 h, whereas anatase-related peaks cannot be seen in the diffraction pattern of Ti/NrGO-800-1h, and this phase presents a crystallite size of ca. 71 nm for Ti/NrGO-800-3h. Interestingly, the TiO_2_-rutile phase presented crystalline domains larger than those of the TiO_2_-anatase for all of the composites, indicating the preferential growth of this phase under these synthesis conditions.

The molar fraction of TiO_2_ phases in the composites was determined according to the Spurr and Myers method (eq 1 and eq 2):^32^
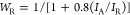
1
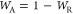
2where *W*_R_ and *W*_A_ are the molar fractions of anatase and rutile TiO_2_ phases, respectively, and *I*_R_ and *I*_A_ are the intensities of the anatase (211) and rutile (001) peaks. The molar fractions of anatase/rutile (*W*_A_/*W*_R_) are given in Table S1. The annealing treatment resulted in a preferential formation of the TiO_2_-rutile phase for all of the synthesized composites. Additionally, the presence of urea favored the formation of rutile since a higher contribution of this phase was observed for the N-doped composite annealed for 1 h (100%) than that of its undoped analog (61%). However, the presence of anatase increased with the annealing duration from 0 to 12%. In this context, the rutile phase is thermodynamically more stable, and its formation is irreversible. Hence, a posterior transformation to anatase during longer treatments cannot explain the results. Thus, the presence of urea seems to play an important role in the titanium phase transformation during the preparation of the composites.^33^

**Figure 2:**
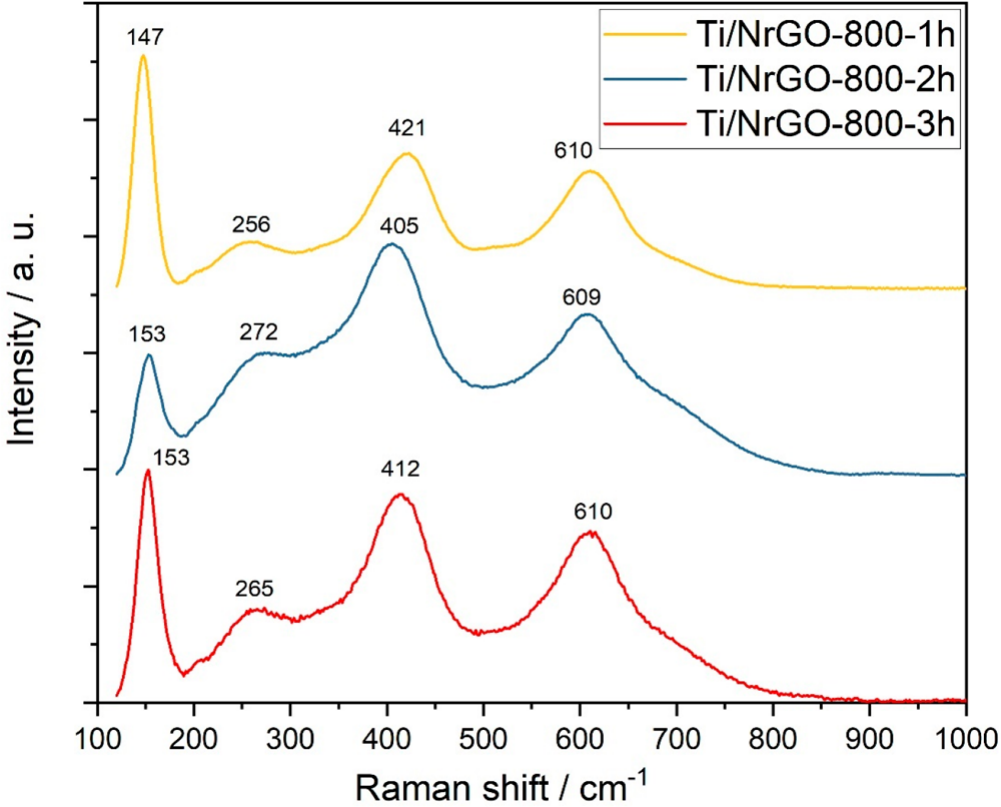
Raman spectra from 100 to 1000 cm^*–*1^ of the N-doped composites obtained at different annealing times.

The structural features of the synthesized composites were further studied using Raman. Figure 2 compares the Raman spectra from 100 to 1000 cm^–1^ of N-doped composites obtained at different annealing times. In all of the cases, a prominent peak around 150 cm^–1^ is evident, which is ascribed to the most intense band of TiO_2_-anatase, whereas the presence of three bands at 260, 410, and 610 cm^–1^ confirms the formation of the rutile phase.^31,33−35^ The characteristic bands of anatase at Raman shifts of 195, 395, 510, and 630 cm^–1^ are not perceived in the spectra of the composites due to their lower relative intensities compared to rutile bands. These results confirm the formation of anatase and rutile phases in all the synthesized composites. In this regard, the presence of anatase-TiO_2_ in Ti/NrGO-800-1h catalyst was not confirmed by XRD, which may be explained by a low content of this phase and/or with a crystallite size below the detection value (<2 nm).

**Figure 3:**
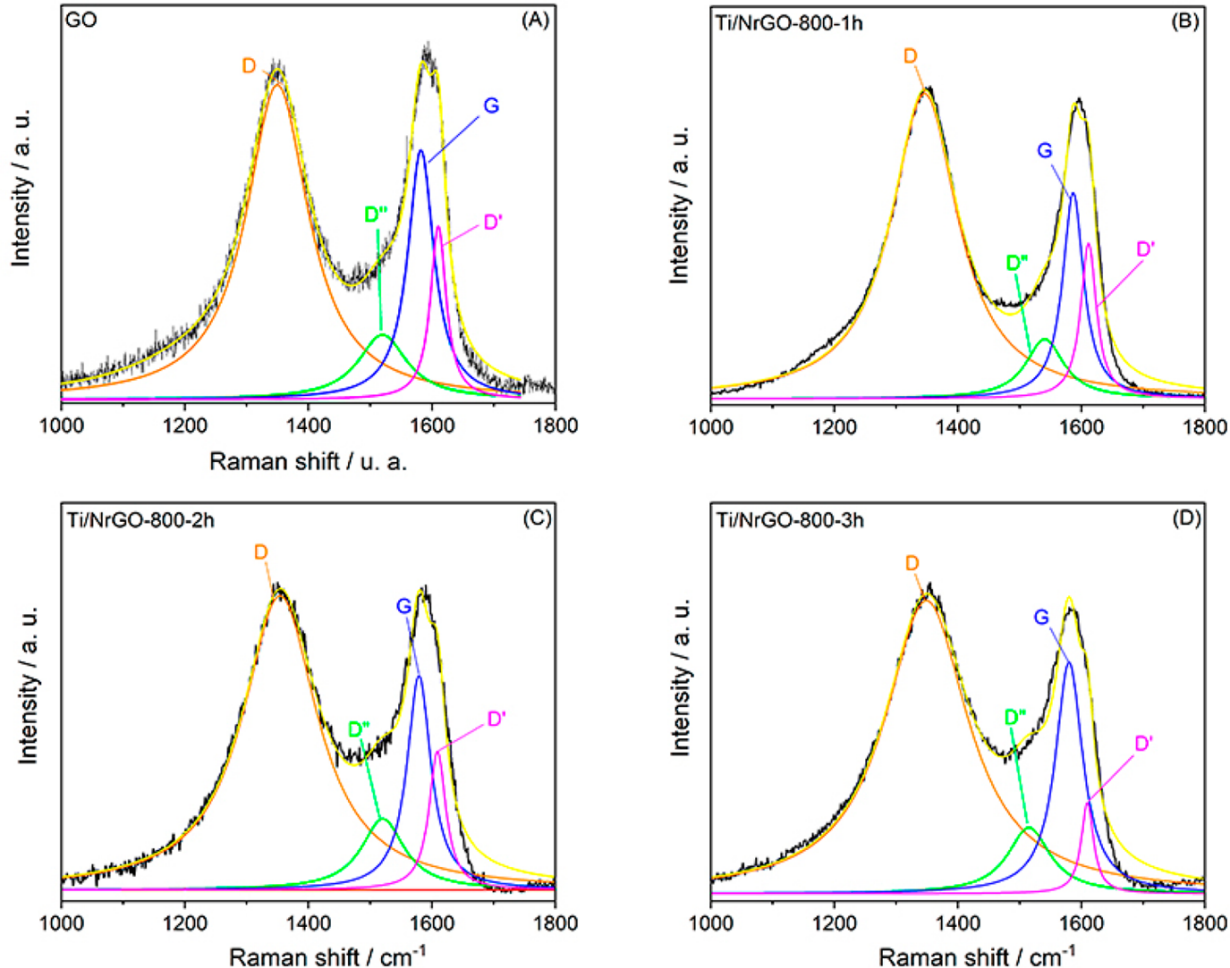
Raman spectra from 1000 to 1800 cm^*–*1^ of (A) GO, (B) Ti/NrGO-800-1h, (C) Ti/NrGO-800-2h, and (D) Ti/NrGO-800-3h.

Raman spectroscopy was also performed to get insights into the graphitic structure of graphene-based composites. Figure 3 shows the deconvolution of Raman spectra of graphene oxide and the N-doped composites obtained at different annealing times in the shift range of 1000–1800 cm^–1^. Raman shift values are summarized in Table S2. Raman spectra were analyzed by peak fitting of the sum of four Lorentzian contributions.^36−39^ The two prominent peaks at 1350–1352 cm^–1^ and 1579–1590 cm^–1^ correspond to D and G bands, which are ascribed to disordered and graphitic ordered structures, respectively. The D band corresponds to the breathing modes of an aromatic ring activated by the presence of a defect,^39−41^ whereas the G band is due to the graphite E_2g_ vibrational mode.^42^ In the G band, a shoulder centered at about 1609 cm^–1^ can be noted corresponding to the D′ band, which is attributed to lattice vibrations involving isolated graphene layers (i.e., those surrounded by intercalation functional groups or oxidized sp^2^ carbon).^39,43,44^ Finally, the D″ band is associated with interstitial defects (opposite to in-plane defects responsible for D).^39,45^

The ratio between the intensities of the D and G bands (*I*_D_/*I*_G_) was determined as an indicator of the defect density in the graphene structure (Table S2).^25,39^ In all of the composites, a decrease in the intensity of the D′ band compared to the starting graphene oxide material was observed. This is related to an effective removal of the oxygenated species intercalated among the graphene layers during the thermal treatment at 800 °C. Despite this, similar *I*_D_/*I*_G_ values were obtained for the TiO_2_-based composites (in the range 1.25–1.47) compared to the starting GO (1.28). The latter can be explained by the incorporation of nitrogen in the graphene framework, which leads to a higher defect density but of a different chemical nature than that of the defects related to oxygenated groups.^46^ In accordance with this, the composite obtained upon annealing for 1 h exhibited the largest nitrogen content (11.7%), and the highest *I*_D_/*I*_G_ ratio was obtained (1.47), while a less effective nitrogen-doping (7 and 2 wt % for Ti/NrGO-800-2h and Ti/NrGO-800-3h, respectively) and, consequently, a lower *I*_D_/*I*_G_ (1.35 and 1.25, for Ti/NrGO-800-2h and Ti/NrGO-800-3h respectively) ratio was obtained as the treatment duration increased.

**Figure 4:**
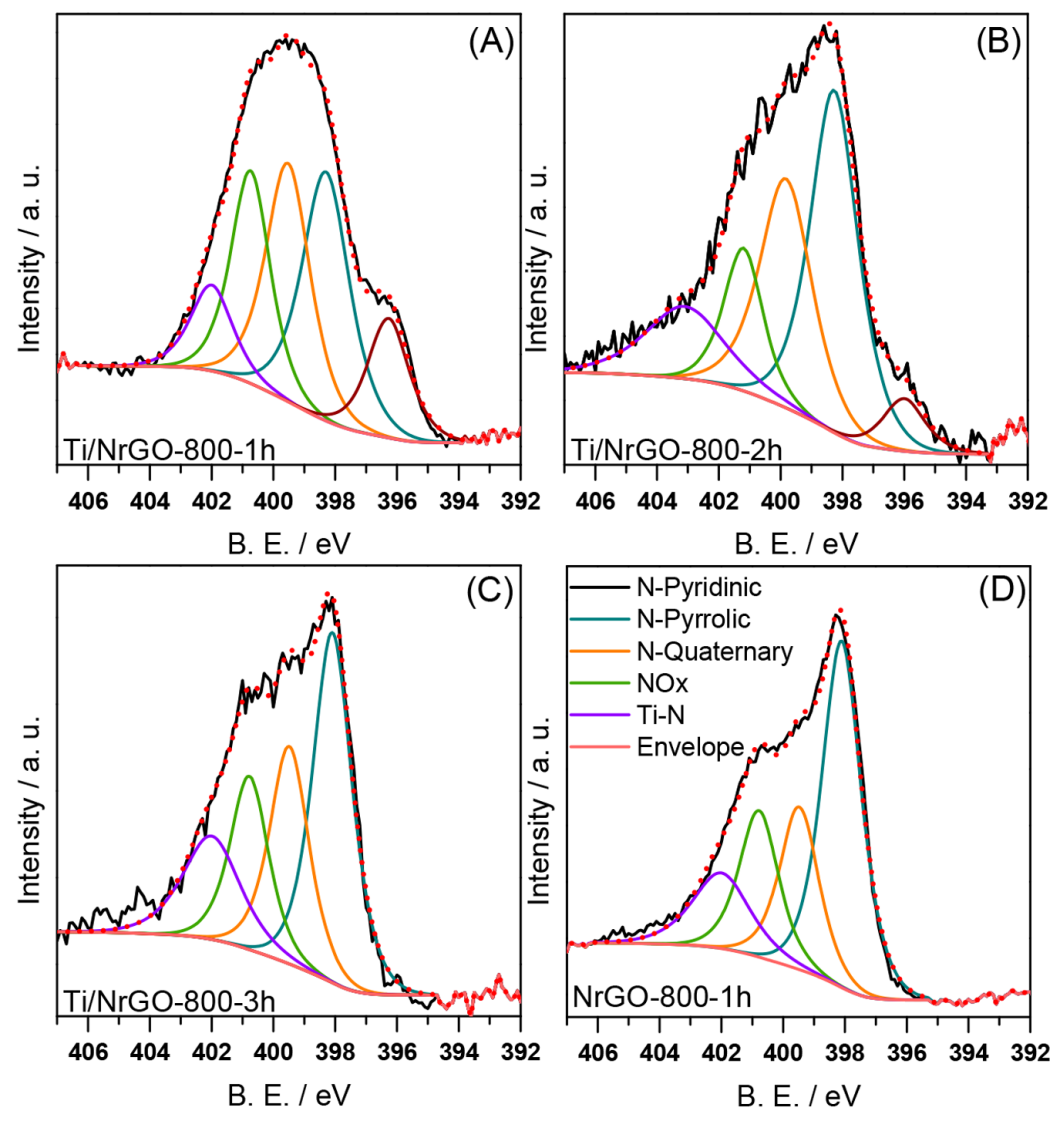
High-resolution N 1s spectra of (A) Ti/NrGO-800-1h, (B) Ti/NrGO-800-2h, (C) Ti/NrGO-800-3h, and (D) NrGO-800-1h.

The surface chemical composition and the ratios C/N and Ti/N were determined from analysis of the XPS spectra (Table S3). The surface nitrogen content obtained by XPS follows a similar trend to the bulk nitrogen determined by elemental analysis, which is longer annealing treatments resulting in a lower introduction of surface nitrogenated species and, consequently, in higher C/N and Ti/N ratios. The nature of the nitrogen species of the composites obtained at different annealing times and the metal-free NrGO-800-1h material was further investigated by XPS. The results obtained from the deconvolution of high resolution N 1s are shown in Figure 4. High resolution N 1s spectra were deconvoluted into four contributions centered at ca. 398, 399, 400, and 402 eV, corresponding to pyridinic N (NII in Table S3), pyrrolic N (NIII in Table S3), quaternary/graphitic N (NIV in Table S3), and N oxides (NV in Table S3), respectively.^46−48^ Also, a peak at ca. 396 eV (NI in Table S3) is evident for Ti/NrGO-800-1h and Ti/NrGO-800-2h composites, which is associated with N–Ti interactions.^49^

The relative content (%) of the corresponding peaks is given in Table S3. All the materials present pyridinic N as the main contribution: the titanium-free material NrGO-800-1h and the composite annealed for 3 h exhibited the highest relative contents of this group (45 and 42%, respectively), whereas a lower value (30–34%) was found for the samples treated for 1 and 2 h. Pyrrolic and graphitic N functionalities are the second and third most common components with similar contributions for all of the synthesized electrocatalysts around 21–27% and 16–21%, respectively. Regarding the effect of the annealing, a progressive increase of NO_*x*_ species was found as the thermal treatment was longer. In the case of the contribution relative to N–Ti interactions, an inverse trend was observed: the NI group decreases as the annealing time increases due to the lower nitrogen content of the composites treated at 800 °C for 2 and 3 h than Ti/NrGO-800-1h. Indeed, the NI group is not evident in the high resolution N 1s spectra of the composite annealed for 3 h.

**Figure 5:**
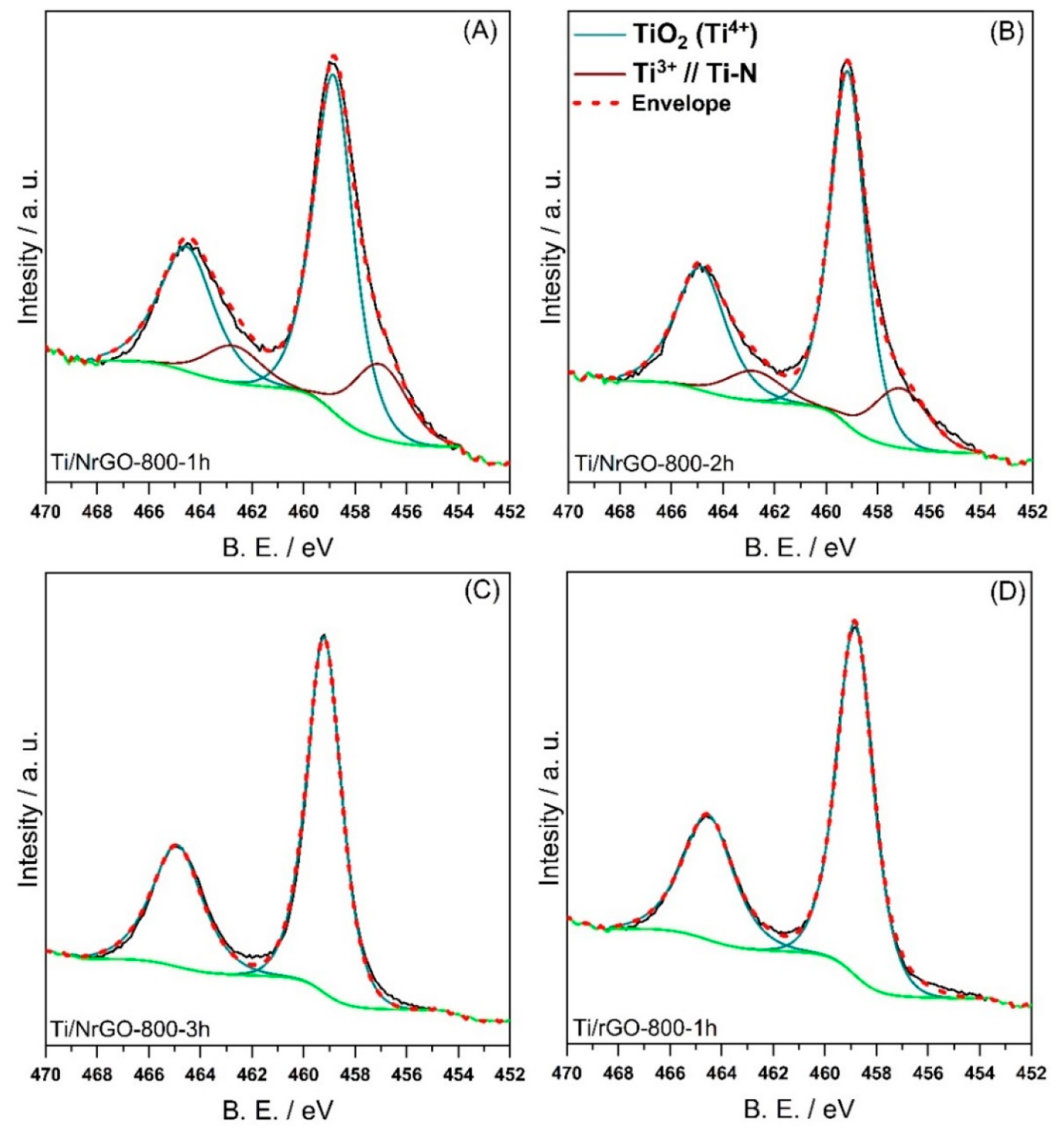
High-resolution Ti 2p spectra of (A) Ti/NrGO-800-1h, (B) Ti/NrGO-800-2h, (C) Ti/NrGO-800-3h, and (D) Ti/rGO-800-1h.

Figure 5 displays the high-resolution Ti 2p spectra of the N-doped composites obtained at different annealing times and the undoped material (Ti/rGO-800-1h). For all of the materials, a spin–orbital doublet appears at ca. 459.0 and 464.8 eV, corresponding to the orbitals Ti 2p_3/2_ and Ti 2p_1/2_, respectively, which is associated with the presence of Ti(IV) (denoted as TiII in Table S3).^18,50,51^ Two peaks located at ca. 457.0 and 462.7 eV are also evident for the N-doped composites annealed for 1 and 2 h, which can be ascribed to Ti–N interactions^49,52^ (TiI in Table S3). According to other authors, this component could also be attributed to the presence of Ti(III).^51^Table S3 evidences a higher contribution of these species (TiI) as the annealing time decreases (i.e., as the nitrogen content increases) in agreement with the results obtained from the high resolution N 1s spectra. Additionally, the composite Ti/NrGO-800-3h again did not present this contribution.

**Figure 6:**
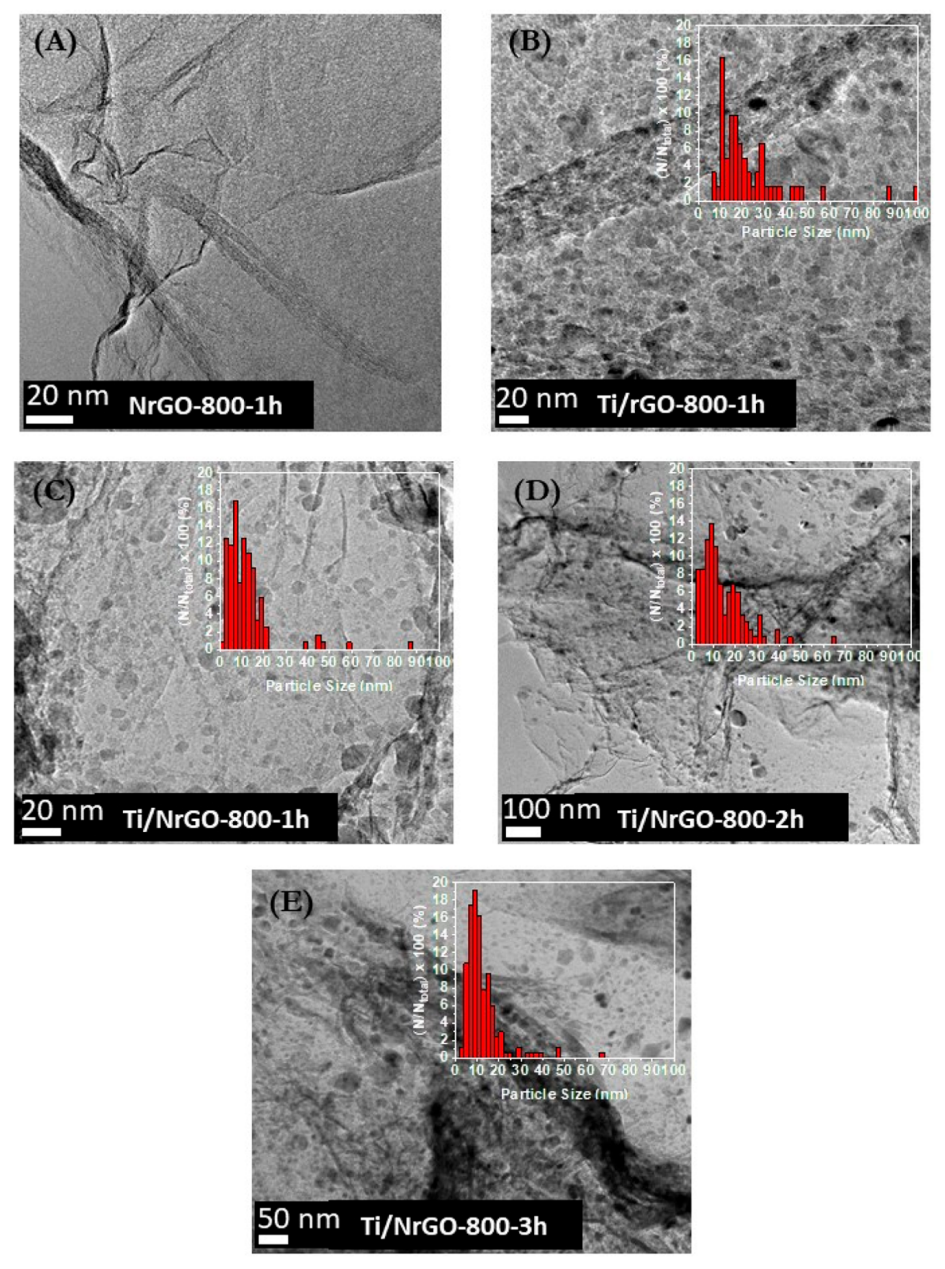
High-resolution TEM images of synthesized materials (A) NrGO-800-1h, (B) Ti/rGO-800-1h, (C) Ti/NrGO-800-1h, (D) Ti/NrGO-800-2h, and (E) Ti/NrGO-800-3h. The insets are metal particle size distribution histograms of the respective catalyst.

TEM images of the composites are shown in Figure 6 (histograms inserted in the images of Ti composites). The graphene sheets with few layers can be seen in the TEM image of NrGO-800-1h. The Ti/rGO-800-1h composite shows a greater heterogeneity of particle sizes ranging from 5 to 100 nm, and a higher agglomeration of the nanoparticles can also be observed. For the Ti and nitrogen composites, similar TEM images were observed. The metal nanoparticles’ dispersion is more uniform than in undoped the Ti composite, but with some agglomerates of nanoparticles.

#### Electrochemical Activity in Alkaline Media

The ORR and OER electrocatalytic activity of Ti/NrGO composites was studied by linear sweep voltammetry (LSV) in 0.1 M NaOH aqueous solution using an RDE or an RRDE as a working electrode.

##### Electrochemical Measurements toward Oxygen Reduction Reaction (ORR)

**Figure 7:**
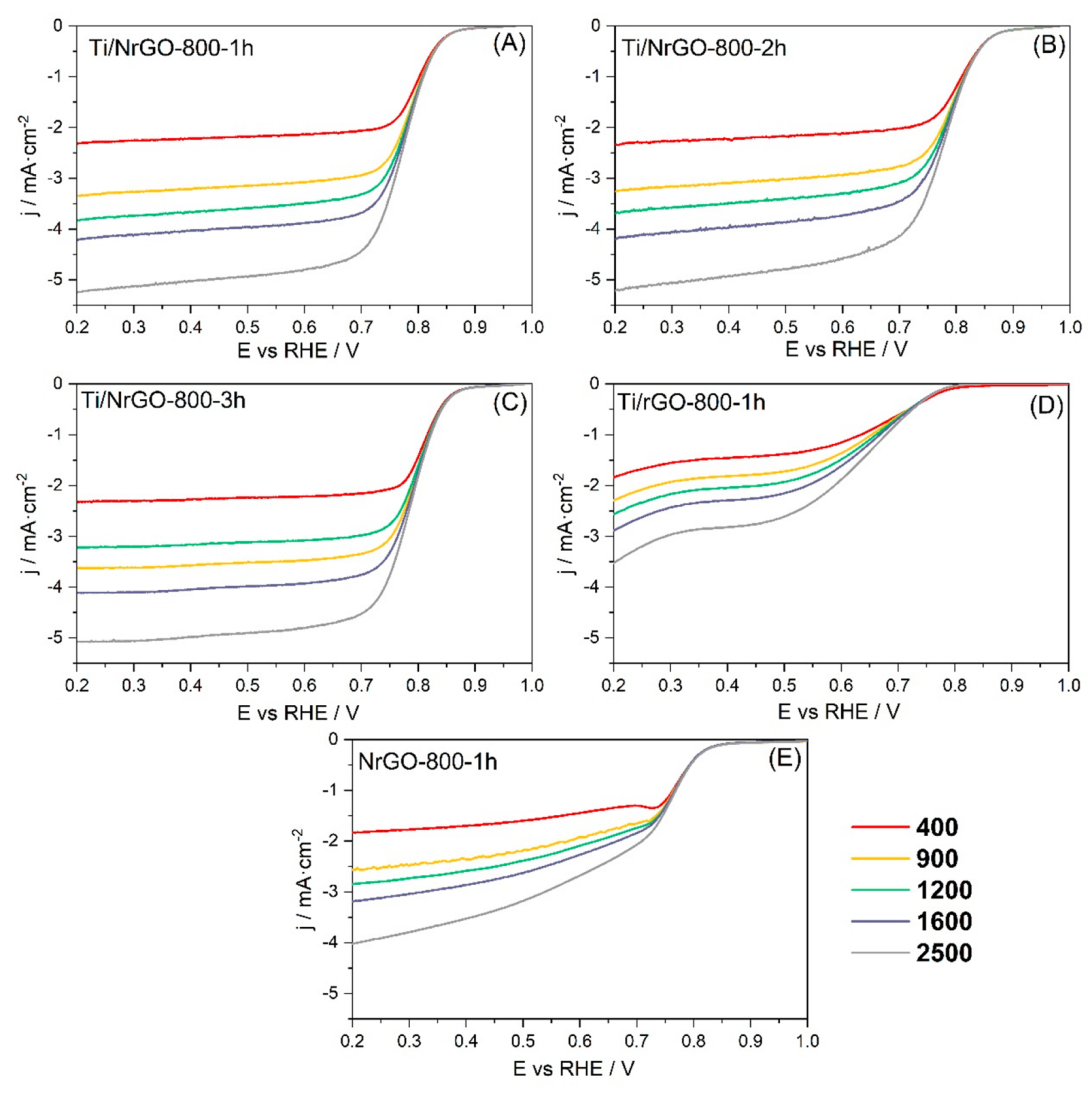
LSV at 0.005 V s^–1^ in an O_2_-saturated 0.1 M NaOH solution, recorded using different electrode rotating rates indicated in the legend (rpm). (A) Ti/NrGO-800-1h, (B) Ti/NrGO-800-2h, (C) Ti/NrGO-800-3h, (D) Ti/rGO-800-1h, (E) NrGO-800-1h.

The polarization curves for the ORR were recorded in an O_2_-saturated alkaline medium.^53,54^ LSV curves at different rotation rates are shown in Figure 7 for the various investigated Ti/NrGO composite catalysts.

The reported ORR current density was determined by subtracting the capacitive contribution from the curves measured in the absence of O_2_. The exchanged number of electrons of each composite was investigated by applying the Koutecky–Levich (K–L) equation (eq 3):
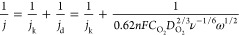
3where *j* is the experimental current density (mA cm^–2^), *j*_k_ is the kinetic current density (mA cm^–2^), *j*_d_ is the diffusion limited current density (mA cm^–2^), *n* is the number of electrons, *F* is the Faraday constant (96 485 C mol^–1^), *C*_O_2__ is the solubility of oxygen in the electrolyte (1.2 × 10^–6^ mol cm^–3^), *D*_O_2__ is the diffusion coefficient of O_2_ in the electrolyte (1.9 × 10^–5^ cm^2^ s^–1^), ν is the kinematic viscosity of 0.1 M NaOH aqueous solution (1.1 × 10^–2^ cm^2^ s^–1^), and ω is the electrode rotation rate (rad s^–1^).

**Figure 8:**
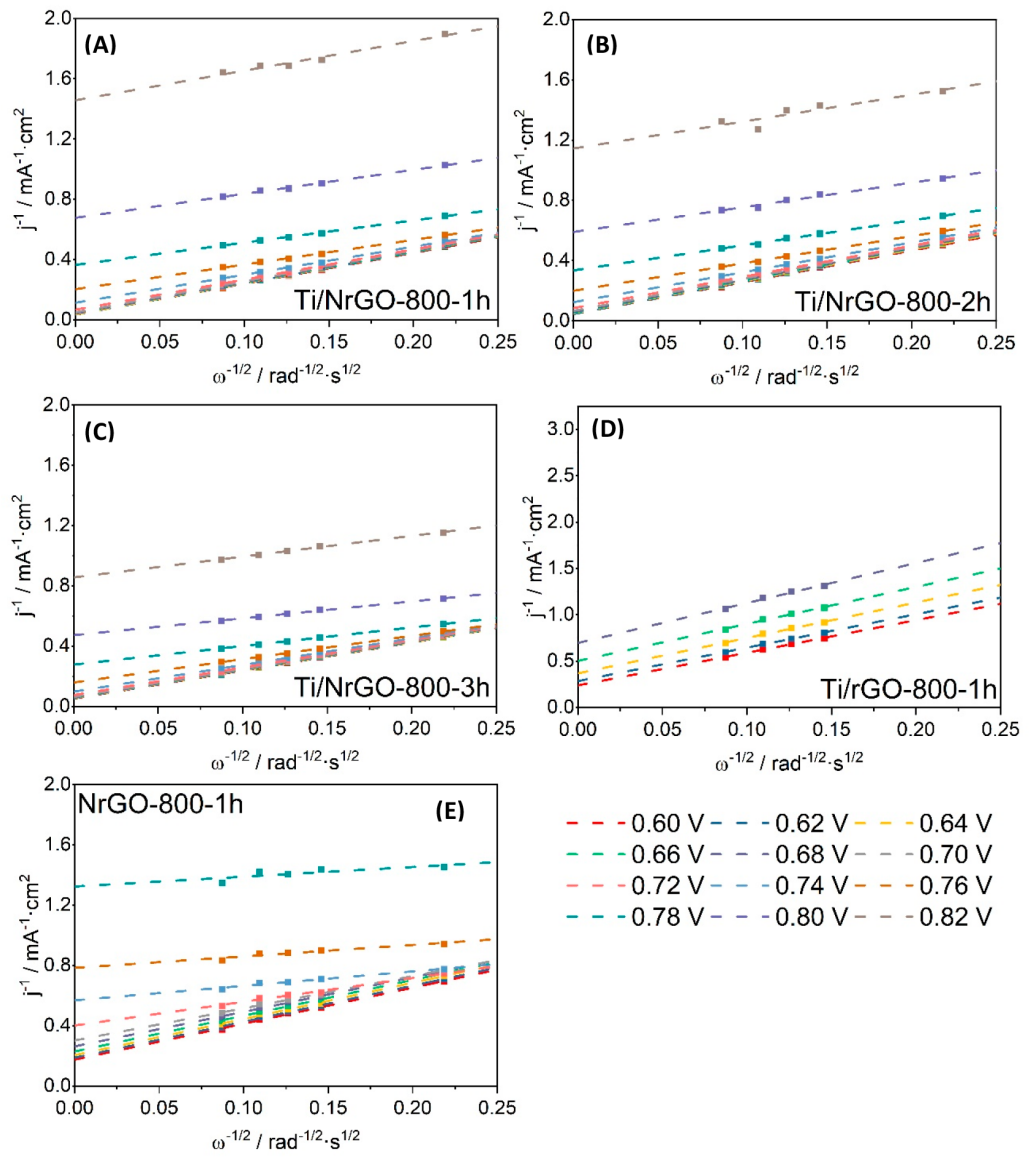
Koutecky–Levich diagrams obtained for each composite. (A) Ti/NrGO-800-1h, (B) Ti/NrGO-800-2h, (C) Ti/NrGO-800-3h, (D) Ti/rGO-800-1h, (E) NrGO-800-1h.

K–L plots are reported in Figure 8 for each composite. The electron transfer number was determined by the linear correlation between the inverse of current density and the inverse of the square root of rotation rate at 0.6 V vs RHE,^55^ as summarized in Table 2. This number (*n*) was found between 2.9 for NrGO-800-1h, 3.3 for Ti/NrGO-800-1h, and 3.5 for both Ti/NrGO-800 composites annealed for 2 and 3 h. According to this, all N-doped composites present a mixed distribution of active sites where some of them proceed through the O_2_ reduction to OH^–^ (4 e^–^ mechanism), while others reduce O_2_ to HO_2_^–^ (less efficient 2 e^–^ mechanism). The higher *n* values of Ti/NrGO-800-2h and Ti/NrGO-800-3h indicate an improved efficiency for oxygen reduction compared to the rest of the catalysts.

**Table 2:** Electrochemical Parameters Obtained from ORR Studies

catalyst	*Ε*_onset_ (V vs RHE)	*E*_1/2_ (V vs RHE)	|*j*_lim=0.3VvsRHE_| (mA·cm^–2^)	*n* (0.6 V)	|*j*_k,*E*=0.82VvsRHE_| (mA·cm^–2^)	% HO_2_^–^*E* = 0.3 V vs RHE
Ti/NrGO-800-1h	0.87	0.78	4.11	3.3	0.67	16
Ti/NrGO-800-2h	0.89	0.79	4.06	3.5	0.87	13
Ti/NrGO-800-3h	0.89	0.79	4.02	3.5	1.17	17
Ti/rGO-800-1h	0.81	0.62	2.42	2.1		7
NrGO-800-1h	0.85	0.72	3.03	2.9		21
Pt/C (40%)	1.01	0.83	5.87	4		<1

In addition, the kinetic current density (*j*_k_) values were determined from K–L plots at different potentials. The *j*_k_ values at 0.82 V vs RHE are reported in Table 2, showing a progressive increase for Ti/NrGO composites with annealing duration. For Ti/rGO-800-1h and NrGO-800-1h composites, it is not possible to make this comparison due to their low activity. These results demonstrate that composite materials with N-doped graphene and Ti oxides have better ORR performance than those phases alone, indicating a positive synergic effect between Ti and the N introduced in the graphene matrix. However, a higher content of nitrogen does not lead to a better electrocatalytic performance since the composite Ti/rGO-800-1h with the highest nitrogen content exhibited the lowest *j*_k_ (0.67 mA cm^–2^).

**Figure 9:**
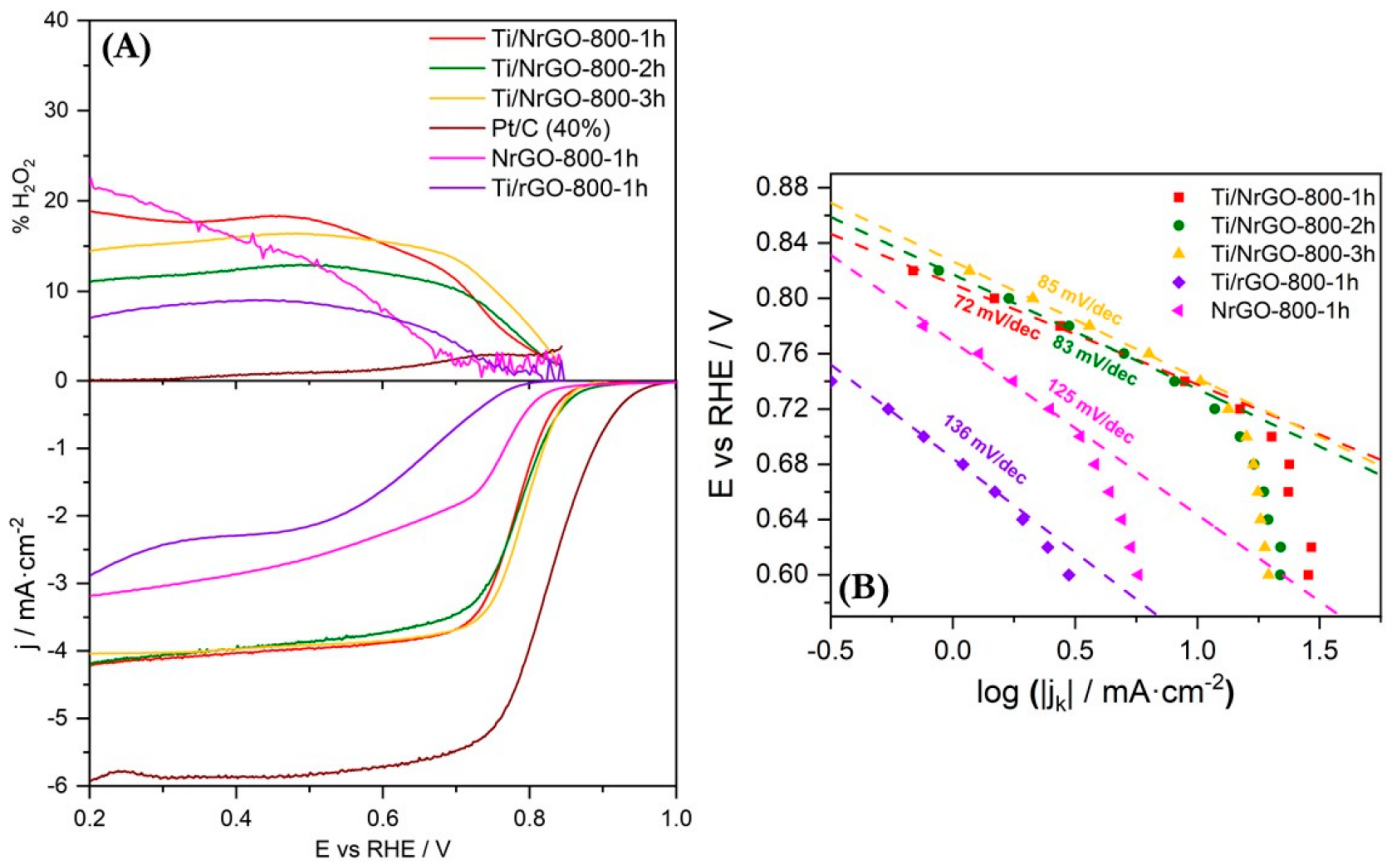
(A) LSV at 0.005 V·s^*–*1^ in an O_2_-saturated 0.1 M NaOH aqueous solution, recorded at 1600 rpm and compared with commercial Pt/C catalyst (40 wt % Pt, Johnson Matthey). Bottom: disk current density. Top: platinum ring signal at 1.2 V vs RHE. (B) Tafel slopes calculated for each composite.

Other relevant electrochemical parameters, such as the onset potential (*E*_onset_) and the half-wave potential (*E*_1/2_), have been studied using a RRDE (Figure 9A and Table 2). The ring potential was set to 1.2 V vs RHE to monitor the formation of HO_2_^–^. Electrochemical results as E_onset_, *E*_1/2_, *j*_lim_, and % HO_2_^–^ obtained from Figure 9A are shown in Table 2.

Ti/NrGO composites have very similar values of *E*_onset_ and *E*_1/2_, with slightly better activity for 2 h and 3 h annealed catalysts. Moreover, current density values at *E* = 0.3 V vs RHE, which can be assumed to be associated with the limiting current density, are also very similar for the three Ti/NrGO composites prepared at different annealing times (around 4 mA·cm^–2^). All of them show lower values than Pt/C commercial catalysts (5.8 mA·cm^–2^), demonstrating a lower amount of 4e^–^ active sites, in agreement with the K–L discussion. The hydrogen peroxide percentage determined from RRDE (% HO_2_^–^) is very similar in Ti/NrGO-800-1h and Ti/NrGO-800-3h, being slightly lower in Ti/NrGO-800-2h. This suggests that the annealing time influences the efficiency of active sites with an optimum duration upon 2 h. On the other hand, the better activity of the composites compared to NrGO-800-1h and Ti/rGO-800-1h suggests that the interaction between Ti and N increases the number of more efficient active sites. By comparing the catalysts annealed for 1 h, the half-wave potential is 60 mV more positive for the Ti/NrGO-800-1h composite than for NrGO-800-1h (without titanium) and 160 mV better than Ti/rGO-800-1h (without nitrogen).

Figure 9B shows Tafel slope values obtained from Figure 9A, bottom. In a previous study, Shinagawa et al. investigated the ORR mechanism and the correlation of rate determining steps with the Tafel slope in alkaline media.^56^ The authors established an associative mechanism as previously described by Adzic et al.^57,58^ In accordance with this mechanism, the global reaction rated at low overpotential can be determined by three main reactions (eqs 4–6):
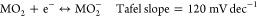
4
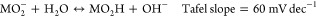
5
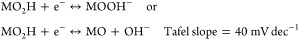
6All Ti/NrGO composites present similar Tafel slope values between 72 and 85 mV·dec^–1^. These values are intermediate between those associated with eqs 4 and 5, being the rate determining step (rds). This indicates that the composites present a mix of different active sites: some of them behave by means of a mechanism where the rds is the adsorption of a hydrogen atom from water on the metal oxide surface (eq 5), very similar to that obtained by a commercial catalyst of Pt/C (60 mV·dec^–1^),^53,58^ and other active sites proceed with the first electron transfer (eq 4), being the rds. The latter reaction appears as the rds for NrGO-800-1h and Ti/rGO-800-1h, since they have Tafel slope values near 120 mV·dec^–1^. These results point to the creation of more active phases upon thermal annealing in the presence of both titanium and nitrogen, which are clearly not formed with the absence of any of these species.

##### Electrochemical Measurements toward Oxygen Evolution Reaction (OER)

**Figure 10:**
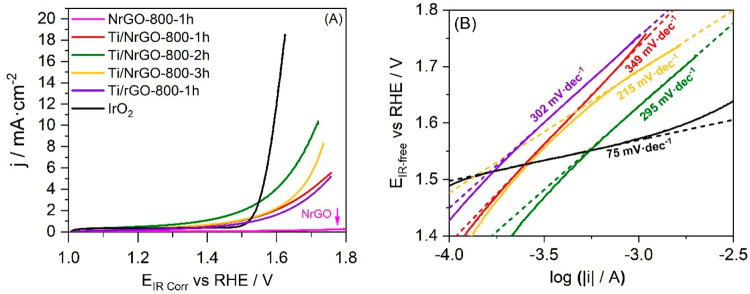
(A) LSV at 0.01 V·s^*–*1^ in a deaerated 0.1 M NaOH aqueous solution, at 1600 rpm to study activity toward OER. (B) Tafel slopes for OER.

The OER activity of synthesized composites has also been measured by LSV and compared with a commercial IrO_2_ catalyst. Figure 10 shows the IR-compensated LSV obtained between 1.0 and 1.8 V vs RHE in deaerated 0.1 M NaOH as an electrolyte.

In order to compare the activity toward OER, the overpotential (η; *E*^0^ = 1.23 V vs RHE) has been calculated at 5 and 10 mA·cm^–2^,^59,60^ as reported in Table 3. It can be observed as the Ti/NrGO-800-2h composite is clearly the most active of synthesized composites, showing the lowest overpotential values. In all cases, the commercial IrO_2_ catalyst shows better activity toward OER than synthesized catalysts, with a higher current at low overpotential.

**Table 3:** Electrochemical Parameters Obtained from OER Studies

catalyst	η (V) |*j*| = 5 mA·cm^–2^	η (V) |*j*| = 10 mA·cm^–2^	Tafel slope (mV·dec^–1^)
Ti/NrGO-800-1h	0.510		349
Ti/NrGO-800-2h	0.400	0.480	295
Ti/NrGO-800-3h	0.460		215
Ti/rGO-800-1h	0.520		302
NrGO-800-1h			
IrO_2_	0.340	0.370	75

According to Shinagawa studies,^56^ the OER main rate-determining step is the following reaction:
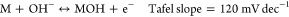
7In line with the theoretical values for the Tafel slope proposed by Shinagawa et al.,^56^ a Tafel slope over 120 mV·dec^–1^ suggests the presence of parallel reactions as carbon oxidation, which makes it not possible to determine the rate-determining step. In any case, the lowest Tafel slope was observed for the catalyst annealed for 3 h, with a progressive and significant decrease of Tafel slope from 349 to 215 mV dec^–1^ with the increase of heat treatment duration.

### Bifunctional Electrocatalytic Activity

The results for the ORR show that N-doped materials in combination with Ti have the best performance as catalysts for this reaction. In addition, it can be observed that the Ti/NrGO-800-2h and Ti/NrGO-800-3h composites present faster kinetics and a higher number of transferred electrons than the others in the series. However, the % HO_2_^–^ is lower in the Ti/NrGO-800-2h catalyst, indicating a better yield for this composite. On the other hand, the catalyst with the best catalytic behavior toward OER was also Ti/NrGO-800-2h, showing higher current density as well as the lowest η value.

**Figure 11:**
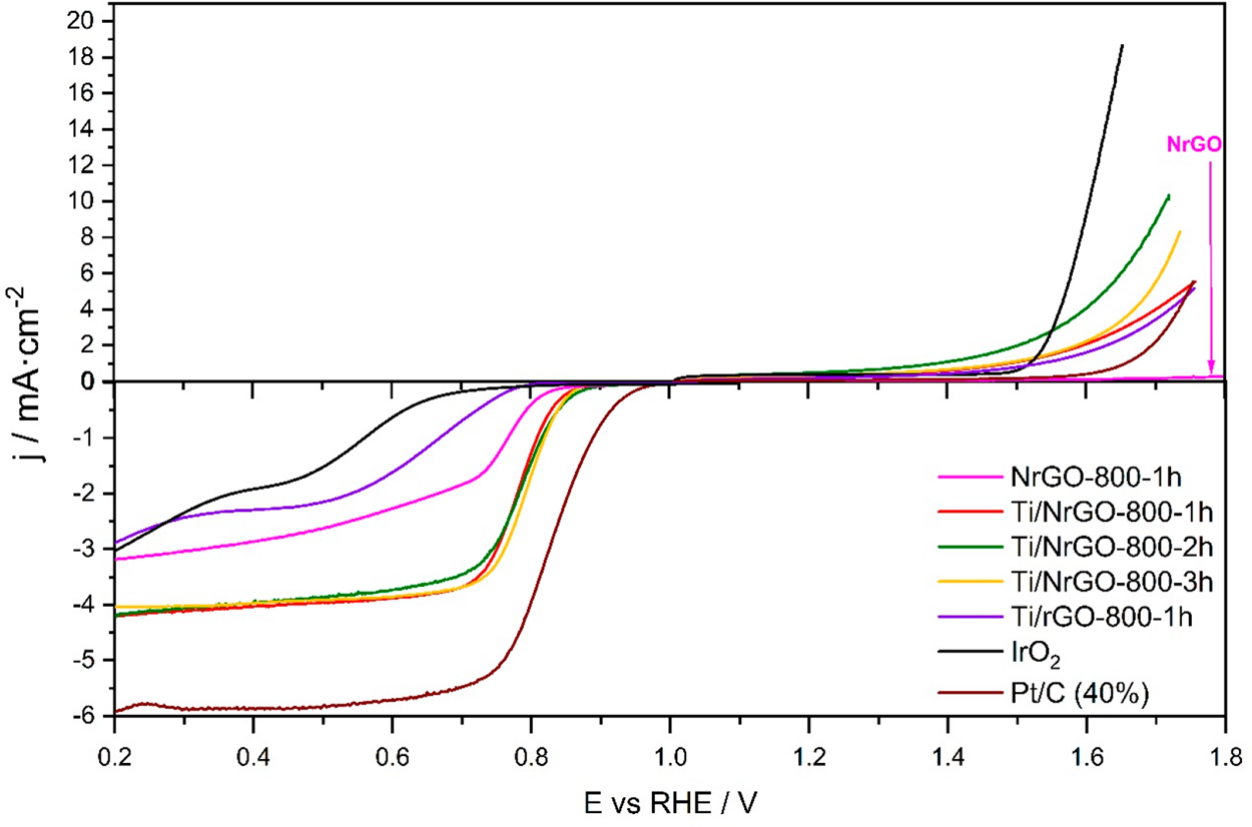
Bifunctional behavior of catalysts obtained by the comparison between ORR (Figure 9A) and OER (Figure 10A) signals.

To better compare and understand the bifunctional catalytic ability, the difference in potential or potential gap (Δ*E*) between the OER current density at 5 or 10 mA cm^–2^ and the ORR half-wave potential (*E*_1/2_) are presented in Table 4. The smaller the difference (Δ*E*), the better is the potential of the electrocatalyst to be used at the oxygen electrode for practical applications in a URFC. Moreover, the oxygen electrode activities for the composites are shown in Figure 11. The bifunctional potential gap (Δ*E*) of the composites follows the order of Ti/NrGO-800-2h < Ti/NrGO-800-3h < Pt/C < Ti/NrGO-800-1h < IrO_2_ < Ti/rGO-800-h < NrGO-800-1h. More significantly, such Δ*E* values for Ti/NrGO-800-2h and Ti/NrGO-800-3h are even smaller than the ones for the noble materials. Therefore, according with these results, Ti/NrGO-800-2h is the catalyst with the best bifunctional ORR/OER behavior. To sum up, in terms of bifunctional behavior for both ORR and OER, a proper optimization requires to take into consideration the activity toward each reaction.

**Table 4:** Comparison of Bifunctional Oxygen Electrode Activity Data in Terms of Potential Difference (Δ*E*) between OER (at 5 or 10 mA cm^–2^, iR-corrected) and ORR (*E*_1/2_)

catalyst	Δ*E* (V) |*j*|_OER_ = 5 mA cm^–2^	Δ*E* (V) |*j*|_OER_ = 10 mA cm^–2^
Ti/NrGO-800-1h	0.960	
Ti/NrGO-800-2h	0.846	0.934
Ti/NrGO-800-3h	0.902	
Ti/rGO-800-1h	1.132	
NrGO-800-1h		
IrO_2_	1.067	1.102
Pt/C (40%)	0.920	

## Discussion

The results obtained reveal a strong dependence on the nitrogen and the titania phase on the structure and catalytic activity for the oxygen electrode in a URFC. Synthesis parameters affect the degree of nitrogen incorporation into the graphene structure and the anatase/rutile ratio. Furthermore, urea, as a nitrogen source, holds an important effect on the formation of TiO_2_/N-doped graphene composites by a sol–gel method and during the pyrolysis procedure with different annealing times.

Once the sol–gel process has been carried out for 12 h, the gel formed from −Ti–O-Ti– species in the presence of graphene oxide and urea is pyrolyzed at 3 °C min^–1^ until 800 °C. In this process, different phenomena take place simultaneously. On the one hand, the thermal decomposition of urea in an inert atmosphere occurs at mild temperatures (below 600 °C), which implies the generation of derivatives such as cyanuric acid, biuret, (HNCO)_*x*_, NH_3_, and others, depending on reaction conditions. In any case, they can work as reducing agents.^61^ On the other hand, there is a generation of the crystalline TiO_2_ phases (anatase–rutile), which depends considerably on the synthesis parameters such as temperature, pH, treatment time, concentrations, dopants such as nitrogen, etc.^33,62^ The XRD data of the undoped catalyst Ti/rGO-800-1h reflects the existence of mixed TiO_2_ anatase and rutile crystalline phases (*W*_A_/*W*_R_ = 39:61). However, the same synthesis conditions in the presence of urea (Ti/NrGO-800-1h) resulted in the formation of only the TiO_2_ rutile phase. This result indicates that the reducing conditions during thermal decomposition of urea promote the formation of rutile. However, the presence of anatase increased with the annealing duration from 0 to 11% and 12% in Ti/NrGO-800-2h and Ti/NrGO-800-3h, respectively. Since the formation of the rutile phase is irreversible, this behavior could be explained assuming that N, the ionic radius of which is only 6% larger than oxygen, interacts with Ti, replacing sublattice oxygen. It is known that N-doping encourages alterations in the surface structure, electronic properties, and defect formation of TiO_2_ phases.^63^ Ti–N interaction has been observed in the Ti/NrGO-800-1h catalyst by XPS, whose contribution decreases with the annealing time, leading to the formation of TiO_2_ anatase while nitrogen is being evaporated. This phenomenon, by contrast, does not occur in the Ti/rGO-800-1h composite, where N’s absence would cause a proper growth of −Ti–O–Ti– structure, giving place first to the anatase structure, which evolves to rutile with the temperature increasing.

Electrochemical results for the ORR show an improvement of the catalytic performance in nitrogen-doped graphene composites compared to the titanium-free or nitrogen-free materials. This behavior can be attributed to different cooperative effects. First, various authors report that Ti^3+^ species could increase the catalytic activity toward ORR, due to the formation of oxygen vacancies (Vo).^14,18^ In Ti/NrGO-800-1h and Ti/NrGO-800-2h composites, the presence of Ti^3+^ or Ti–N species are confirmed by XPS. In the case of Ti/NrGO-800-3h, the larger crystalline domains of TiO_2_ phases and lower amount of nitrogen, makes it more difficult to determine Ti^3+^ or Ti–N species by XPS. On the other hand, it is known that N species associated with nitrogen-doped graphene structures are active for ORR, especially N-pyridinic species, which are observed in Ti/NrGO composites.^46,64^ This can explain the good ORR performance of these composites compared to Ti/rGO-800-1h. However, it is observed that the increase of the annealing time does not significantly affect the activity, which suggests that the nitrogen amount does not play an important role in the performance of the ORR.

For the OER performance, the Ti-free composite (NrGO-800-1h) displays the lowest activity, indicating that titanium oxide is essential for the formation of active sites. Unlike the ORR, the annealing time in the composite formation is significant for the oxygen evolution reaction. The composite annealed over 2 h shows the lower overpotential for OER. Similar to ORR, Ti^3+^ can provide active sites for water adsorption and dissociation, which is a critical first step in OER.^19^ Additionally, the N-doped graphene structure can have abundant active sites and functional groups to efficiently increase the OER.^19^ Moreover, the interactions between rutile and anatase phases is a much more decisive factor in the OER than in the ORR. According to Hu et al.,^22^ the electrons transfer from rutile to anatase, and the holes transfer in the reverse direction. This is due to the fact that the energy of the conduction band of rutile is higher than that of anatase. Therefore, the positively charged rutile reduces the activation energy, making it easier to adsorb hydroxyl anions. Among Ti/NrGO composites, those annealed at 2 and 3 h show the higher anatase/rutile ratio. However, the lower amount of nitrogen in Ti/NrGO-800-3h makes it less active than Ti/NrGO-800-2h catalysts.

Overall, Ti/NrGO-800-2h is the most active bifunctional catalyst, which appears to be due to a combination of the following factors: (a) anatase/rutile interaction, (b) N-doping graphene, and (c) the presence of Ti^3+^ and/or Ti–N species. However, it should be noted that, in terms of bifunctional behavior for both ORR and OER, a proper optimization requires taking into consideration the activity toward each reaction, and in particular to OER.

## Conclusions

A method is herein described for the preparation of TiO_2_/N-doped graphene composites, which are active for both ORR and OER in alkaline media. The benefits of this synthetic method include simplicity, control over the anatase/rutile ratio, and the incorporation of nitrogen atoms in the graphene structure. We have discovered that an insulating material such as TiO_2_ can be active in two electrochemical reactions for the oxygen electrode, thanks to features such as control of the anatase/rutile interaction, N-doping graphene, and the formation of Ti^3+^/Ti–N species. This research can afford a new strategy for tailoring more efficient Ti-based electrocatalysts.

## Supporting Information Available

The binding energies are given in brackets. The Supporting Information is available free of charge at https://pubs.acs.org/doi/10.1021/acs.iecr.1c02896.

Crystallite sizes (nm), lattice parameters, number of graphene layers and molar ratio of TiO_2_ phases (%) from XRD patterns; Raman shift (cm^–1^) of the G, D, D′, and D″ from Raman spectra deconvolution; surface carbon, oxygen, nitrogen, and titanium contents and C/N and Ti/N ratios calculated by XPS of the electrocatalysts and the relative content (%) of the deconvoluted peaks of N 1s and Ti 2p (PDF)

## References

[ref1] Hydrogen Roadmap Europe; Fuel Cells and Hydrogen Joint Undertaking (FCH): Brussels, Belgium, 2019, 10.2843/249013.

[ref2] Pettersson J.; Ramsey B.; Harrison D. A Review of the Latest Developments in Electrodes for Unitised Regenerative Polymer Electrolyte Fuel Cells. J. Power Sources 2006, 157 (1), 28–34. 10.1016/j.jpowsour.2006.01.059.

[ref3] Bhogilla S. S.; Ito H.; Kato A.; Nakano A. Research and Development of a Laboratory Scale Totalized Hydrogen Energy Utilization System. Int. J. Hydrogen Energy 2016, 41 (2), 1224–1236. 10.1016/j.ijhydene.2015.10.105.

[ref4] Zhao S.; Yan L.; Luo H.; Mustain W.; Xu H. Recent Progress and Perspectives of Bifunctional Oxygen Reduction/Evolution Catalyst Development for Regenerative Anion Exchange Membrane Fuel Cells. Nano Energy; Elsevier Ltd., 2018; pp 172–198.

[ref5] Wang Y.-J.; Fang B.; Wang X.; Ignaszak A.; Liu Y.; Li A.; Zhang L.; Zhang J. Recent Advancements in the Development of Bifunctional Electrocatalysts for Oxygen Electrodes in Unitized Regenerative Fuel Cells (URFCs). Prog. Mater. Sci. 2018, 98, 108–167. 10.1016/j.pmatsci.2018.06.001.

[ref6] Desmond Ng J. W.; Gorlin Y.; Hatsukade T.; Jaramillo T. F. A Precious-Metal-Free Regenerative Fuel Cell for Storing Renewable Electricity. Adv. Energy Mater. 2013, 3 (12), 1545–1550. 10.1002/aenm.201300492.

[ref7] García G.; Roca-Ayats M.; Lillo A.; Galante J. L.; Peña M. A.; Martínez-Huerta M. V. Catalyst Support Effects at the Oxygen Electrode of Unitized Regenerative Fuel Cells. Catal. Today 2013, 210, 67–74. 10.1016/j.cattod.2013.02.003.

[ref8] Roca-Ayats M.; Herreros E.; García G.; Peña M. A.; Martínez-Huerta M. V. Promotion of Oxygen Reduction and Water Oxidation at Pt-Based Electrocatalysts by Titanium Carbonitride. Appl. Catal., B 2016, 183, 53 10.1016/j.apcatb.2015.10.009.

[ref9] Ruiz-Cornejo J. C.; Sebastián D.; Martínez-Huerta M. V.; Lázaro M. J. Tantalum-Based Electrocatalysts Prepared by a Microemulsion Method for the Oxygen Reduction and Evolution Reactions. Electrochim. Acta 2019, 317, 261–271. 10.1016/j.electacta.2019.05.145.

[ref10] Alegre C.; Modica E.; Di Blasi A.; Di Blasi O.; Busacca C.; Ferraro M.; Aricò A. S.; Antonucci V.; Baglio V. NiCo-Loaded Carbon Nanofibers Obtained by Electrospinning: Bifunctional Behavior as Air Electrodes. Renewable Energy 2018, 125, 250–259. 10.1016/j.renene.2018.02.089.

[ref11] McKerracher R. D.; Figueredo-Rodríguez H. A.; Ponce de León C.; Alegre C.; Baglio V.; Aricò A. S.; Walsh F. C. A High-Performance, Bifunctional Oxygen Electrode Catalysed with Palladium and Nickel-Iron Hexacyanoferrate. Electrochim. Acta 2016, 206, 127–133. 10.1016/j.electacta.2016.04.090.

[ref12] Zeng K.; Zheng X.; Li C.; Yan J.; Tian J.; Jin C.; Strasser P.; Yang R. Recent Advances in Non-Noble Bifunctional Oxygen Electrocatalysts toward Large-Scale Production. Adv. Funct. Mater. 2020, 30 (27), 2000503 10.1002/adfm.202000503.

[ref13] Luque-Centeno J. M.; Martínez-Huerta M. V.; Sebastián D.; Pardo J. I.; Lázaro M. J. CoTiO3/NrGO Nanocomposites for Oxygen Evolution and Oxygen Reduction Reactions: Synthesis and Electrocatalytic Performance. Electrochim. Acta 2020, 331, 135396 10.1016/j.electacta.2019.135396.

[ref14] Pei D.; Gong L.; Zhang X.; Chen J.; Mu Y.; Zhang A.; Yu H. Defective Titanium Dioxide Single Crystals Exposed by High-Energy {001} Facets for Efficient Oxygen Reduction. Nat. Commun. 2015, 6, 2–11. 10.1038/ncomms9696. PMC4846326 26493365

[ref15] Zheng R.; Shu C.; Hou Z.; Hu A.; Hei P.; Yang T.; Li J.; Liang R.; Long J. In Situ Fabricating Oxygen Vacancy-Rich TiO2 Nanoparticles via Utilizing Thermodynamically Metastable Ti Atoms on Ti3C2Tx MXene Nanosheet Surface To Boost Electrocatalytic Activity for High-Performance Li–O2 Batteries. ACS Appl. Mater. Interfaces 2019, 11 (50), 46696–46704. 10.1021/acsami.9b14783. 31755689

[ref16] Liu G.; Li W.; Bi R.; Atangana Etogo C.; Yu X.-Y.; Zhang L. Cation-Assisted Formation of Porous TiO2–x Nanoboxes with High Grain Boundary Density as Efficient Electrocatalysts for Lithium–Oxygen Batteries. ACS Catal. 2018, 8 (3), 1720–1727. 10.1021/acscatal.7b04182.

[ref17] Chevallier L.; Bauer A.; Cavaliere S.; Hui R.; Rozière J.; Jones D. Mesoporous Nanostructured Nb-Doped Titanium Dioxide Microsphere Catalyst Supports for PEM Fuel Cell Electrodes. ACS Appl. Mater. Interfaces 2012, 4, 1752 10.1021/am300002j. 22428619

[ref18] Boppella R.; Lee J.-E.; Mota F. M.; Kim J. Y.; Feng Z.; Kim D. H. Composite Hollow Nanostructures Composed of Carbon-Coated Ti3+ Self-Doped TiO2-Reduced Graphene Oxide as an Efficient Electrocatalyst for Oxygen Reduction. J. Mater. Chem. A 2017, 5 (15), 7072–7080. 10.1039/C7TA00583K.

[ref19] He L.; Liu J.; Liu Y.; Cui B.; Hu B.; Wang M.; Tian K.; Song Y.; Wu S.; Zhang Z.; et al. Titanium Dioxide Encapsulated Carbon-Nitride Nanosheets Derived from MXene and Melamine-Cyanuric Acid Composite as a Multifunctional Electrocatalyst for Hydrogen and Oxygen Evolution Reaction and Oxygen Reduction Reaction. Appl. Catal., B 2019, 248, 366–379. 10.1016/j.apcatb.2019.02.033.

[ref20] Jin S.; Li C.; Shrestha L. K.; Yamauchi Y.; Ariga K.; Hill J. P. Simple Fabrication of Titanium Dioxide/N-Doped Carbon Hybrid Material as Non-Precious Metal Electrocatalyst for the Oxygen Reduction Reaction. ACS Appl. Mater. Interfaces 2017, 9 (22), 18782–18789. 10.1021/acsami.7b03305. 28481078

[ref21] Shan Z.; Archana P. S.; Shen G.; Gupta A.; Bakker M. G.; Pan S. NanoCOT: Low-Cost Nanostructured Electrode Containing Carbon, Oxygen, and Titanium for Efficient Oxygen Evolution Reaction. J. Am. Chem. Soc. 2015, 137 (37), 11996–12005. 10.1021/jacs.5b05367. 26340536

[ref22] Hu Y.; Ding T.; Zhang K.; Li B.; Zhu B.; Tang K. Component-Tunable Rutile-Anatase TiO2/Reduced Graphene Oxide Nanocomposites for Enhancement of Electrocatalytic Oxygen Evolution. ChemNanoMat 2018, 4 (11), 1133–1139. 10.1002/cnma.201800252.

[ref23] Luque-Centeno J. M.; Martínez-Huerta M. V.; Sebastián D.; Lemes G.; Pastor E.; Lázaro M. J. Bifunctional N-Doped Graphene Ti and Co Nanocomposites for the Oxygen Reduction and Evolution Reactions. Renewable Energy 2018, 125, 182–192. 10.1016/j.renene.2018.02.073.

[ref24] Hummers W. S.; Offeman R. E. Preparation of Graphitic Oxide. J. Am. Chem. Soc. 1958, 80 (6), 1339 10.1021/ja01539a017.

[ref25] Kaniyoor A.; Baby T. T.; Arockiadoss T.; Rajalakshmi N.; Ramaprabhu S. Wrinkled Graphenes: A Study on the Effects of Synthesis Parameters on Exfoliation-Reduction of Graphite Oxide. J. Phys. Chem. C 2011, 115 (36), 17660–17669. 10.1021/jp204039k.

[ref26] Pfanzelt M.; Kubiak P.; Fleischhammer M.; Wohlfahrt-Mehrens M. TiO2 Rutile—An Alternative Anode Material for Safe Lithium-Ion Batteries. J. Power Sources 2011, 196 (16), 6815–6821. 10.1016/j.jpowsour.2010.09.109.

[ref27] Dhandole L. K.; Ryu J.; Lim J.-M.; Oh B.-T.; Park J. H.; Kim B.-G.; Jang J. S. Hydrothermal Synthesis of Titanate Nanotubes from TiO2 Nanorods Prepared via a Molten Salt Flux Method as an Effective Adsorbent for Strontium Ion Recovery. RSC Adv. 2016, 6 (100), 98449–98456. 10.1039/C6RA14769K.

[ref28] Fuel Cell Handbook (Seventh ed.); EG&G Technical Services: Morgantown, WV, 2004; pp 26507–0880.

[ref29] Verma R.; Samdarshi S. K.; Sagar K.; Konwar B. K. Nanostructured Bi-Phasic TiO2 Nanoparticles Grown on Reduced Graphene Oxide with High Visible Light Photocatalytic Detoxification. Mater. Chem. Phys. 2017, 186, 202–211. 10.1016/j.matchemphys.2016.10.045.

[ref30] Pumera M. Electrochemistry of Graphene: New Horizons for Sensing and Energy Storage. Chem. Rec. 2009, 9 (4), 211–223. 10.1002/tcr.200900008. 19739147

[ref31] Khalifa Z. S. Grain Size Reduction on Nanostructured TiO2 Thin Films Due to Annealing. RSC Adv. 2017, 7 (48), 30295–30302. 10.1039/C7RA00706J.

[ref32] Spurr R. A.; Myers H. Quantitative Analysis of Anatase-Rutile Mixtures with an X-Ray Diffractometer. Anal. Chem. 1957, 29 (5), 760–762. 10.1021/ac60125a006.

[ref33] Hanaor D. A. H.; Sorrell C. C. Review of the Anatase to Rutile Phase Transformation. J. Mater. Sci. 2011, 46 (4), 855–874. 10.1007/s10853-010-5113-0.

[ref34] Ma H. L.; Yang J. Y.; Dai Y.; Zhang Y. B.; Lu B.; Ma G. H. Raman Study of Phase Transformation of TiO 2 Rutile Single Crystal Irradiated by Infrared Femtosecond Laser. Appl. Surf. Sci. 2007, 253 (18), 7497–7500. 10.1016/j.apsusc.2007.03.047.

[ref35] Martinez-Huerta M. V.; Fierro J. L. G.; Banares M. Monitoring the States of Vanadium Oxide during the Transformation of TiO 2 Anatase-to-Rutile under Reactive Environments: H 2 Reduction and Oxidative Dehydrogenation of Ethane. Catal. Commun. 2009, 11, 15 10.1016/j.catcom.2009.08.002.

[ref36] Antunes E. F.; Lobo A. O.; Corat E. J.; Trava-Airoldi V. J.; Martin A. A.; Veríssimo C. Comparative Study of First- and Second-Order Raman Spectra of MWCNT at Visible and Infrared Laser Excitation. Carbon 2006, 44 (11), 2202–2211. 10.1016/j.carbon.2006.03.003.

[ref37] Wang L.; Zhao J.; Sun Y. Y.; Zhang S. B. Characteristics of Raman Spectra for Graphene Oxide from Ab Initio Simulations. J. Chem. Phys. 2011, 135 (18), 184503 10.1063/1.3658859. 22088071

[ref38] Hawaldar R.; Merino P.; Correia M. R.; Bdikin I.; Gracio J.; Mendez J.; Martin-Gago J. A.; Singh M. K. Large-Area High-Throughput Synthesis of Monolayer Graphene Sheet by Hot Filament Thermal Chemical Vapor Deposition. Sci. Rep. 2012, 2, 682 10.1038/srep00682. 23002423 PMC3448070

[ref39] Torres D.; Pinilla J. L.; Moliner R.; Suelves I. On the Oxidation Degree of Few-Layer Graphene Oxide Sheets Obtained from Chemically Oxidized Multiwall Carbon Nanotubes. Carbon 2015, 81, 405–417. 10.1016/j.carbon.2014.09.073.

[ref40] Ferrari A. C.; Robertson J. Interpretation of Raman Spectra of Disordered and Amorphous Carbon. Phys. Rev. B: Condens. Matter Mater. Phys. 2000, 61 (20), 14095–14107. 10.1103/PhysRevB.61.14095.

[ref41] Ferrari A. C. Raman Spectroscopy of Graphene and Graphite: Disorder, Electron–Phonon Coupling, Doping and Nonadiabatic Effects. Solid State Commun. 2007, 143 (1–2), 47–57. 10.1016/j.ssc.2007.03.052.

[ref42] Saito R.; Hofmann M.; Dresselhaus G.; Jorio A.; Dresselhaus M. S. Raman Spectroscopy of Graphene and Carbon Nanotubes. Adv. Phys. 2011, 60 (3), 413–550. 10.1080/00018732.2011.582251.

[ref43] Cuesta A.; Dhamelincourt P.; Laureyns J.; Martínez-Alonso A.; Tascón J. M. D. Raman Microprobe Studies on Carbon Materials. Carbon 1994, 32 (8), 1523–1532. 10.1016/0008-6223(94)90148-1.

[ref44] Nakamizo M.; Tamai K. Raman Spectra of the Oxidized and Polished Surfaces of Carbon. Carbon 1984, 22 (2), 197–198. 10.1016/0008-6223(84)90216-1.

[ref45] Jawhari T.; Roid A.; Casado J. Raman Spectroscopic Characterization of Some Commercially Available Carbon Black Materials. Carbon 1995, 33 (11), 1561–1565. 10.1016/0008-6223(95)00117-V.

[ref46] Lemes G.; Sebastián D.; Pastor E.; Lázaro M. J. N-Doped Graphene Catalysts with High Nitrogen Concentration for the Oxygen Reduction Reaction. J. Power Sources 2019, 438, 227036 10.1016/j.jpowsour.2019.227036.

[ref47] Sheng Z.-H.; Shao L.; Chen J.-J.; Bao W.-J.; Wang F.-B.; Xia X.-H. Catalyst-Free Synthesis of Nitrogen-Doped Graphene via Thermal Annealing Graphite Oxide with Melamine and Its Excellent Electrocatalysis. ACS Nano 2011, 5 (6), 4350–4358. 10.1021/nn103584t. 21574601

[ref48] Liu J.; Zhang T.; Wang Z.; Dawson G.; Chen W. Simple Pyrolysis of Urea into Graphitic Carbon Nitride with Recyclable Adsorption and Photocatalytic Activity. J. Mater. Chem. 2011, 21 (38), 14398 10.1039/c1jm12620b.

[ref49] Fechler N.; Fellinger T.-P.; Antonietti M. Template-Free One-Pot Synthesis of Porous Binary and Ternary Metal Nitride@N-Doped Carbon Composites from Ionic Liquids. Chem. Mater. 2012, 24 (4), 713–719. 10.1021/cm203667g.

[ref50] Deshmukh S. P.; Kale D. P.; Kar S.; Shirsath S. R.; Bhanvase B. A.; Saharan V. K.; Sonawane S. H. Ultrasound Assisted Preparation of RGO/TiO2 Nanocomposite for Effective Photocatalytic Degradation of Methylene Blue under Sunlight. Nano-Structures & Nano-Objects 2020, 21, 100407 10.1016/j.nanoso.2019.100407.

[ref51] Bellamkonda S.; Thangavel N.; Hafeez H. Y.; Neppolian B.; Ranga Rao G. Highly Active and Stable Multi-Walled Carbon Nanotubes-Graphene-TiO2 Nanohybrid: An Efficient Non-Noble Metal Photocatalyst for Water Splitting. Catal. Today 2019, 321–322, 120–127. 10.1016/j.cattod.2017.10.023.

[ref52] Wang Y.; Li L.; An C.; Wang Y.; Chen C.; Jiao L.; Yuan H. Facile Synthesis of TiN Decorated Graphene and Its Enhanced Catalytic Effects on Dehydrogenation Performance of Magnesium Hydride. Nanoscale 2014, 6 (12), 6684–6691. 10.1039/c4nr00474d. 24817573

[ref53] Spendelow J. S.; Wieckowski A. Electrocatalysis of Oxygen Reduction and Small Alcohol Oxidation in Alkaline Media. Phys. Chem. Chem. Phys. 2007, 9 (21), 2654–2675. 10.1039/b703315j. 17627310

[ref54] Markovic N. M.; Ross P. N. Surface Science Studies of Model Fuel Cell Electrocatalysts. Surf. Sci. Rep. 2002, 45 (4–6), 117–229. 10.1016/S0167-5729(01)00022-X.

[ref55] Xing W.; Yin G.; Zhang J. Rotating Electrode Methods and Oxygen Reduction Electrocatalysts; Elsevier, 2014.

[ref56] Shinagawa T.; Garcia-Esparza A. T.; Takanabe K. Insight on Tafel Slopes from a Microkinetic Analysis of Aqueous Electrocatalysis for Energy Conversion. Sci. Rep. 2015, 5, 13801 10.1038/srep13801. 26348156 PMC4642571

[ref57] Shao M.; Liu P.; Adzic R. R. Superoxide Anion Is the Intermediate in the Oxygen Reduction Reaction on Platinum Electrodes. J. Am. Chem. Soc. 2006, 128 (23), 7408–7409. 10.1021/ja061246s. 16756272

[ref58] Paulus U. A.; Schmidt T. J.; Gasteiger H. A.; Behm R. J. Oxygen Reduction on a High-Surface Area Pt/Vulcan Carbon Catalyst: A Thin-Film Rotating Ring-Disk Electrode Study. J. Electroanal. Chem. 2001, 495 (2), 134–145. 10.1016/S0022-0728(00)00407-1.

[ref59] Moriau L. J.; Bele M.; Vižintin A.; Ruiz-Zepeda F.; Petek U.; Jovanovič P.; Šala M.; Gaberšček M.; Hodnik N. Synthesis and Advanced Electrochemical Characterization of Multifunctional Electrocatalytic Composite for Unitized Regenerative Fuel Cell. ACS Catal. 2019, 9 (12), 11468–11483. 10.1021/acscatal.9b03385.

[ref60] Gorlin Y.; Jaramillo T. F. A Bifunctional Nonprecious Metal Catalyst for Oxygen Reduction and Water Oxidation. J. Am. Chem. Soc. 2010, 132 (39), 13612–13614. 10.1021/ja104587v. 20839797

[ref61] Wakeland S.; Martinez R.; Grey J. K.; Luhrs C. C. Production of Graphene from Graphite Oxide Using Urea as Expansion-Reduction Agent. Carbon 2010, 48 (12), 3463–3470. 10.1016/j.carbon.2010.05.043.

[ref62] Miszczak S.; Pietrzyk B. Anatase–Rutile Transformation of TiO2 Sol–Gel Coatings Deposited on Different Substrates. Ceram. Int. 2015, 41 (6), 7461–7465. 10.1016/j.ceramint.2015.02.066.

[ref63] Batzill M.; Morales E. H.; Diebold U. Influence of Nitrogen Doping on the Defect Formation and Surface Properties of TiO2 Rutile and Anatase. Phys. Rev. Lett. 2006, 96 (2), 1–4. 10.1103/PhysRevLett.96.026103. 16486602

[ref64] Quilez-Bermejo J.; Morallon E.; Cazorla-Amoros D. Oxygen-Reduction Catalysis of N-Doped Carbons Prepared via Heat Treatment of Polyaniline at over 1100 Degrees C. Chem. Commun. 2018, 54 (35), 4441–4444. 10.1039/C8CC02105H. 29651494

