# Supplementary material for: Titanium Dioxide/N-Doped Graphene Composites as Non-Noble Bifunctional Oxygen Electrocatalysts
Source: Ind Eng Chem Res. 2021 Nov 19;60(51):18817–30. doi: 10.1021/acs.iecr.1c02896 (PMC9134212; doi:10.1021/acs.iecr.1c02896)
Supplement: Supplementary file 1 — ie1c02896_si_001.pdf [file ie1c02896_si_001.pdf]

Supporting Information

Titanium dioxide/N-doped graphene composites as non-noble bifunctional oxygen electrocatalysts

José Manuel Luque-Centeno [a,b], María Victoria Martínez-Huerta [a*], David Sebastián [b], Sara Pérez-Rodríguez [b], María Jesús Lázaro [b]

[a] Instituto de Catálisis y Petroleoquímica (CSIC), Marie Curie 2, 28049, Madrid, Spain

[b] Instituto de Carboquímica (CSIC), Miguel Luesma Castán 4, 50018, Zaragoza, Spain

*Corresponding author: mmartinez@icp.csic.es

Table S1. Crystallite sizes (nm), lattice parameters (a and c for rutile and anatase, c for carbon, in Angstroms), number of graphene layers and molar ratio of TiO_2 phases (%) from XRD patterns.

Material	Rutile-TiO_2 (nm)	Anatase-TiO_2 (nm)	Carbon (nm)	N	W_A:W_R
**ICDD reference**	a=4.584<br>c=2.953	a=3.777<br>c=9.501	c=6.7244	-	-
**Ti/NrGO-800-1h**	68<br>a=4.597<br>c=2.961	-	2.7<br>c=6.790	9	0:100
**Ti/NrGO-800-2h**	71<br>a=4.595<br>c=2.960	11.8<br>a=3.788<br>c=9.529	3.3<br>c=6.813	11	11:89
**Ti/NrGO-800-3h**	161<br>a=4.595<br>c=2.960	71<br>a=3.785<br>c=9.519	2.2<br>c=6.7961	7	12:88
**Ti/rGO-800-1h**	11.7<br>a=4.597<br>c=2.964	2.2<br>a=3.783<br>c=9.491	2.1<br>c=6.843	7	39:61
**NrGO-800-1h**	-	-	4.4<br>c=6.727	14	-

Table S2. Raman shift (cm^{-1}) of the G, D, D' and D" from Raman spectra deconvolution.

**Material**	**G**	**D**	**D'**	**D"**	**I_D/I_G**
**GO**	1583	1349	1611	1533	1.28
**Ti/NrGO-800-1h**	1586	1346	1611	1539	1.47
**Ti/NrGO-800-2h**	1579	1353	1608	1520	1.35
**Ti/NrGO-800-3h**	1579	1350	1609	1515	1.25

Table S3. Surface carbon, oxygen, nitrogen and titanium contents and C/N and Ti/N ratios calculated by XPS of the electrocatalysts and the relative content (%) of the deconvoluted peaks of N1s and Ti2p. The binding energies are given in brackets.

Material	XPS (at.%)				Atomic ratios		N1s (%)					Ti2p 3/2 (%)	
	C	O	N	Ti	C/N	Ti/N	NI	NII	NIII	NIV	NV	TiI	TiII
**Ti/NrGO-800-1h**	62	15	7	16	10.3	0.6	13 (396.3)	30 (398.3)	27 (399.5)	21 (400.7)	9 (402.0)	23 (457.0)	77 (458.8)
**Ti/NrGO-800-2h**	71	11	4	14	18.9	0.9	10 (396.8)	34 (398.3)	26 (399.5)	16 (400.8)	14 (402.0)	20 (457.1)	80 (459.2)
**Ti/NrGO-800-3h**	63	15	3	19	23.5	1.7	-	42 (398.2)	21 (399.6)	20 (408)	15 (402.1)	-	100 (459.2)
**Ti/rGO-800-1h**	76	13	-	12	-	-	-	-	-	-	-	-	100 (459.2)
**NrGO-800-1h**	88	5	8	-	13.6	-	-	45 (398.2)	21 (399.6)	19 (400.8)	14 (402.1)	-	-
